# Watch-and-Wait strategy for locally advanced rectal cancer after neoadjuvant chemoradiotherapy: a comprehensive review

**DOI:** 10.3389/fonc.2026.1760042

**Published:** 2026-02-19

**Authors:** Peng Huang, Xiaoyue Zhao, Huanhuan Fei, Linzhu Zhang, Xiaofan Liu, Yongjun Yu, Rong Wu, Fei Fei

**Affiliations:** 1 Nankai University School of Medicine, Nankai University, Tianjin, China; 2 Department of Oncology, Affiliated Jinling Hospital of Medical School of Nanjing University, Nanjing, Jiangsu, China; 3 Department of Gastroenterology, Nanjing First Hospital, Nanjing Medical University, Nanjing, Jiangsu, China; 4 Department of Oncology, Nanjing First Hospital, Nanjing Medical University, Nanjing, Jiangsu, China; 5 Department of Oncology, The First Affiliated Hospital of Nanjing Medical University, Nanjing, Jiangsu, China; 6 Department of Colorectal Surgery, Tianjin Union Medical Center, The First Affiliated Hospital of Nankai University, Tianjin, China; 7 Department of Scientific and Technology, Nanjing First Hospital, Nanjing Medical University, Nanjing, Jiangsu, China

**Keywords:** clinical complete response, locally advanced rectal cancer, MRI, neoadjuvant therapy, Watch-and-Wait strategy

## Abstract

The conventional treatment for locally advanced rectal cancer (LARC) primarily involves neoadjuvant chemoradiotherapy (nCRT) combined with total mesorectal excision (TME). However, surgery-related complications and long-term functional impairments can significantly compromise patients’ quality of life. The watch-and-wait (W&W) strategy has emerged as a non-surgical alternative for patients achieving a clinical complete response (cCR), with advantages in organ preservation. Its safety and efficacy have been validated by multiple clinical studies. Literature retrieval was performed in PubMed (2020–2025), including reviews, RCTs/cohort studies on LARC W&W and cCR/pCR. This comprehensive review summarizes the clinical evidence, patient selection criteria, efficacy assessment methods, challenges, and future directions of the W&W strategy. Based on the latest research, when strictly selecting cCR patients (especially those with sustained cCR after neoadjuvant therapy), the 5-year disease-free survival (DFS) rate of the W&W strategy is comparable to that of the surgical group (70%-85%), with a local regrowth rate of approximately 20%-30%, which can mostly be controlled by salvage surgery. The combination of magnetic resonance imaging (MRI) and circulating tumor DNA (ctDNA) analysis significantly improves the accuracy of cCR assessment. Furthermore, integrating immunotherapy with total neoadjuvant therapy (TNT) has expanded the eligible population for the W&W strategy. This review also highlights current limitations of the W&W strategy, such as the lack of standardized assessment procedures, validated biomarkers, and long-term follow-up data. It proposes that future efforts should focus on multi-center randomized controlled trials and artificial intelligence-assisted assessment models to promote the advancement of the W&W strategy toward precision and standardization.

## 1 Introduction

Colorectal cancer (CRC) is the third most commonly diagnosed cancer and the second leading cause of cancer-related mortality worldwide (1). Rectal cancer accounts for approximately one-third of all CRC cases, with a global incidence rate of 13.9 and 8.6 per 100,000 in males and females, respectively (2).The standard treatment for locally advanced rectal cancer (LARC) is neoadjuvant chemoradiotherapy (nCRT) followed by total mesorectal excision (TME) (3).

Studies have shown that 10%-35% of patients with LARC who receive nCRT achieve pathological complete response (pCR), and these patients exhibit favorable survival outcomes (4), but these patients may face the risk of overtreatment if they undergo surgery (5). However, this treatment regimen is associated with considerable morbidity, including anastomotic leakage, perioperative mortality, and urinary, sexual, and anal sphincter dysfunction (2, 6). Therefore, to avoid perioperative complications and enhance patients’ quality of life, a novel organ-preserving treatment concept—the “Watch-and-Wait (W&W)” strategy after nCRT—has emerged as a new therapeutic option for LARC patients (7–9).

Early data from the International Watch-and-Wait Database (IWWD) showed that the 5-year overall survival (OS) rate of patients on the W&W strategy reached 85%, the disease-specific survival rate was 94%, the local regrowth rate was approximately 25%, and over 80% of cases with local regrowth achieved R0 resection through salvage surgery (10–12). Since it is difficult to predict pCR preoperatively, the selection of the “Watch-and-Wait” strategy currently relies on clinical complete response (cCR). Specifically, after receiving nCRT, LARC patients undergo clinical response assessment using digital rectal examination, endoscopy, and MRI (13). Therefore, further exploration is still needed regarding how to accurately predict pCR, screen the optimal population eligible for the “Watch-and-Wait” strategy, and determine the timing for implementing the “Watch-and-Wait” strategy.

This article aims to systematically integrate high-quality clinical studies published in recent years, comprehensively elaborate on the clinical evidence, patient selection criteria, and efficacy assessment technologies of the W&W strategy, analyze the current challenges, and explore the application prospects of new technologies, thereby providing evidence-based support for clinical practice.

## 2 Clinical evidence and efficacy analysis of the W&W strategy

### 2.1 Efficacy comparison of key clinical studies

An analysis of 793 patients in the IWWD confirmed a 3-year local regrowth-free survival rate of 74.3% and a 5-year distant metastasis-free survival rate of 88.9% (12). Notably, patients maintaining cCR for the first year had an 88.1% probability of remaining regrowth-free in the subsequent two years, rising to 97.3% if cCR was maintained for three years, indicating that patients with long-term cCR have a significantly reduced risk of recurrence (12).

Multiple randomized trials have verified the non-inferiority of the W&W strategy. In the OPRA trial, 324 LARC patients were randomly assigned to receive either induction chemotherapy followed by chemoradiotherapy (INCT-CRT) or chemoradiotherapy followed by consolidation chemotherapy (CRT-CNCT). The W&W strategy was adopted for patients achieving clinical or near-complete response (cCR/nCR). The 3-year TME-free survival rates in the two groups were 41% and 53%, respectively, and the 3-year disease-free survival (DFS) rates were both 76%, comparable to that of the historical surgical control group (14).

A pooled analysis of the CAO/ARO/AIO-12 and OPRA trials revealed no significant difference in DFS between the two TNT strategies (15). If organ preservation is the primary goal, CRT-CNCT should be prioritized due to its higher pCR/cCR rate (15). Another pooled analysis also found that there were no significant differences in the rates of DFS, distant recurrence-free survival (DRFS), local recurrence-free survival (LRFS), and OS among LARC patients who underwent mandatory TME or selected the W&W strategy after TNT, indicating no difference in tumor prognosis (16).

LARC patients with deficient mismatch repair (dMMR)/high microsatellite instability (MSI-H) account for approximately 5% of all LARC patients (17). For these patients, immunotherapy exhibits excellent efficacy, significantly improving the complete response (CR) rate and providing additional theoretical support for the W&W strategy (18).

A multi-center cohort study conducted by Yang et al. showed that among 20 dMMR/MSI-H patients who received neoadjuvant therapy with PD-1 inhibitors, 90% achieved CR, with all seven patients opting for W&W remaining recurrence-free during follow-up (19). The TORCH trial demonstrated that immunotherapy could also enable a significant proportion of proficient MMR (pMMR) patients to achieve CR (54.2%-56.5%), suggesting its applicability beyond pMMR populations (20).

### 2.2 Characteristics of local regrowth and salvage therapy

Local regrowth occurs in approximately 25% of W&W patients (12), predominantly within the first two years (93.7% of cases, median time of 9 months) (6). A secondary analysis of the OPRA trial further indicated that the 2-year local regrowth rate was 24.4% in patients with cCR and 36.6% in patients with nCR, suggesting that the quality of initial response is associated with the risk of regrowth (13). Fernandez et al. found that the risk of local regrowth was 5% or lower in patients who maintained cCR for 3 years, and the risk of systemic recurrence thereafter was less than 2%. Therefore, if rectal cancer patients treated with the W&W strategy achieve and maintain cCR within the first 3 years of initiating the strategy, the intensity of surveillance can be reduced (12).

The feasibility and efficacy of salvage surgery for patients with local regrowth are crucial for the safety of the W&W strategy. Studies demonstrate that over 94% of patients with regrowth undergo salvage surgery (TME or local excision), achieving a 2-year local recurrence-free rate of 97.8% and a disease-free survival rate of 90.3%, comparable to outcomes in the initial surgical group (6). The OPRA trial confirmed a 5-year DFS rate of 70% after salvage TME, not significantly different from the 71% rate in the primary surgery group (14).These data indicate that even in the event of local regrowth, timely salvage surgery can still achieve favorable tumor control without increasing procedural complexity or postoperative morbidity (21).

## 3 Patient selection criteria for the W&W strategy

### 3.1 Definition and assessment criteria of clinical complete response

**Figure 1:**
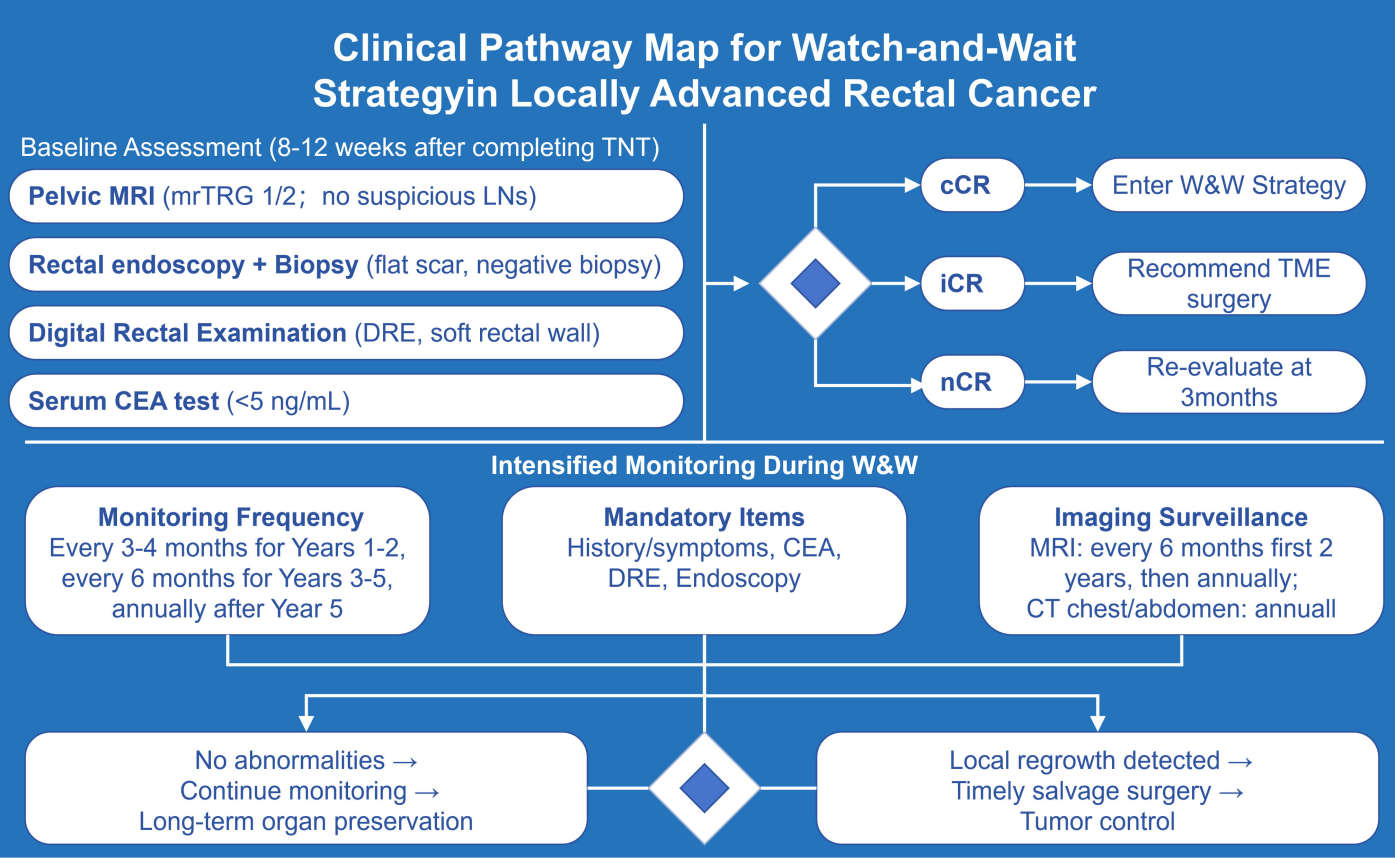
Proposed integrated pathway for patient selection and management in the W&W strategy for LARC.

cCR serves as the foundation for screening patients eligible for the W&W strategy, and its definition requires a comprehensive assessment integrating clinical examination, endoscopy, imaging, and pathological biopsy (**Figure 1**). Currently, internationally recognized cCR criteria include: (1) Digital Rectal Examination (DRE): No tumor is palpable, and the rectal wall is soft without nodules or induration; (2) Endoscopic examination: The original tumor site shows only a flat white scar with telangiectasia, without ulcers, nodules, or masses, and pathological biopsy results are negative; (3) Imaging examination: MRI shows uniform low T2 signal (fibrosis) in the original tumor area, no restricted diffusion on diffusion-weighted imaging (DWI), and regional lymph nodes with a short-axis diameter < 5 mm and regular morphology; (4) Tumor markers: The level of carcinoembryonic antigen (CEA) decreases to the normal range (< 5 ng/mL) (3).

There are slight differences in the detailed definitions of cCR among different guidelines. For example, the Memorial Sloan Kettering Cancer Center (MSKCC) criteria emphasize no residual tumor signal on MRI plus negative endoscopic biopsy, while the Chinese Watch-and-Wait Database (CWWD) criteria additionally include normal CEA and normal DRE findings (3). Pei et al. found that the two criteria exhibited comparable diagnostic performance in predicting cCR among patients receiving nCRT (3). Moreover, these two diagnostic criteria are also applicable to the patient population receiving nCRT combined with immunotherapy.

### 3.2 Impact of neoadjuvant therapy regimens on screening

Total neoadjuvant therapy (TNT) significantly affect the cCR rate, thereby influencing the screening of patients eligible for the W&W strategy. Regimens like INCT-CRT or CRT-CNCT improve tumor regression. In different trials, variations in neoadjuvant therapy regimens have led to differences in cCR and pCR rates. Common trials include the PRODIGE-23 trial (22), RAPIDO trial (23), CAO/ARO/AIO-12 trial (24), and LARCT-US trial (25) (**Table 1**). Notably, compared with the experimental group in the RAPIDO trial, the LARCT-US/AdmL trial involved patients with more advanced tumors and shorter chemotherapy cycles, yet achieved comparable tumor-killing efficacy (25). A trial comparing long-course radiotherapy (LCCRT) and short-course radiotherapy (SCRT) showed that among W&W patients, the cCR rates of LCCRT and SCRT were similar, but patients who received LCCRT had a higher 2-year organ preservation rate and a lower 2-year local regrowth rate (26).

**Table 1:** Summary table of core data from trials on neoadjuvant therapy for LARC.

Trial name	Publication year	Number of patients	Inclusion criteria	Treatment regimen	DFS	TME-free survival	pCR	cCR	OS
Garcia-Aguilar et al (OPRA Trial) (14)	2022	324 cases	Stage II/III	INCT-CRT : INCT→ CRT→ TME/WW	3-year: 76% in both groups	3-year: INCT-CRT :41% CRT-CNCT:53%	Not specified	Not specified	Not specified
				CRT-CNCT : CRT → CNCT→ TME/WW					
Fokas et al (CAO/ARO/AIO-12 Trial) (24)	2019	311 cases (306 evaluable)	≤ 12 cm ; cT3b+/cT4/lymph node involvement	INCT-CRT : 3 FOLFOX → CRT → TME	3-year: INCT-CRT :73.1% CRT-CNCT:72.7% (log-rank p=0.7)	Not mentioned	INCT-CRT:17% CRT-CNCT:25%	Not specified	Not specified
				CRT-CNCT: CRT → 3 FOLFOX → TME					
Gani et al (CAO/ARO/AIO-16 Trial) (31)	2025	93 cases (91 started chemoradiotherapy)	cT1-2N1-2/cT3a-dN0/N1-2; ≤ 12 cm	CRT → 3 FOLFOX → WW/local excision for patients with cCR/nCR, TME for others	3-year: 65%	3-year: 21%	26%	37%	3-year: 94%
Conroy et al (PRODIGE 23 Trial) (22)	2021	461 cases	cT3/cT4M0	Neoadjuvant group : 6 FOLFIRINOX → CRT → TME → 3m adjuvant chemotherapy	3-year: neoadjuvant group : 76% standard group : 69%	Not mentioned	neoadjuvant group : 28%; standard group : 12%	Not specified	Not mentioned
				Standard group: CRT → TME → 6m adjuvant chemotherapy					
Bahadoer et al (RAPIDO Trial) (23)	2021	920 cases (912 eligible)	LARC high-risk on MRI	Experimental group: SCRT→ CAPOX/FOLFOX → TME	Not specified	Not mentioned	experimental group : 28% ; standard group :14%	Not specified	year: experimental group : 89.1% standard group : 88.8%
				Standard group: LCCRT → TME ± adjuvant chemotherapy					
Bercz et al Trial (26)	2024	323 cases	LARC	LCCRT group: LCCRT + CAPOX/FOLFOX	year: 90% in both groups	2-year: LCCRT group : 40% SCRT group : 31%	Not specified	LCCRT: 44.5% SCRT : 43.4%	2-year: LCCRT : 99% SCRT :100%
				SCRT group: SCRT + CAPOX/FOLFOX; cCR(W&W)					
Glimelius et al (LARCT-US Trial) (25)	2024	462 cases ( LARCT-US 273 + AdmL 189)	LARC cT4, cN2, EMVI, MRF+, lateral lymph nodes (enlarged)	SCRT→4 CAPOX / 6 FOLFOX-6 →cCR (W&W), others (TME)	year DrTF: LARCT-US group :28% AdmL group :26%	3-year LARCT-US group :14% AdmL group : 13%	LARCT-US group : 34 (12.5%) AdmL group : 27 (14.3%)	LARCT-US : 31 (11.3%) AdmL : 17 (9.0%)	3-year OS: LARCT-US : 88% AdmL :89%

Furthermore, the combination of immunotherapy with TNT has further increased the cCR rate (**Table 2**). The TORCH trial showed that PD-1 inhibitor plus TNT achieved a CR rate of 54–57% in pMMR/MSS patients, which was significantly higher than that in the traditional TNT group (20). Li et al. also found that combined treatment with SCRT and immune checkpoint inhibitors (ICIs) can achieve more effective tumor regression. This may be attributed to the synergistic effects of radiotherapy and ICIs, such as enhanced antigen release and increased infiltration of tumor-infiltrating lymphocytes (7).

**Table 2:** Summary of trial data on neoadjuvant immunotherapy for LARC.

Trial Name	Publication Year	Patients (Total/Evaluable)	Inclusion Criteria	Treatment Regimen	DFS	cCR	pCR	OS	Adverse Events (Main Grade ≥3)	Other Endpoints
Yu et al. (NEOCAP) (70)	2024	53/52	dMMR/MSI-H or POLE/POLD1-mutated LARC Rectum: cT3 or N+; Colon: cT3 with invasion ≥5mm or T4±N+	Camrelizumab (200mg IV on Day 1) + apatinib (250mg PO on Days 1-14), q3w for 3-6 months; WW for patients with cCR	Median follow-up of 16.4 months; Not mature (specific DFS not reported)	54% (28/52); 24 cases chose WW without recurrence	61% in surgery group (14/23)	Median follow-up of 16.4 months; 1 death from treatment-related hepatitis; OS of others not mature	Grade ≥3 AEs: 38% (20/53)	ORR: 92%; R0 resection rate: 96.7% (22/23)
Xia et al. (TORCH) (20)	2024	130/121	pMMR/MSS LARC cT3-4 or N1 Tumor ≤12cm from anal verge	Group A: SCRT→ 6 CAPOX + toripalimab Group B: 2 CAPOX + toripalimab → SCRT → 4 CAPOX + toripalimab	Median follow-up of 19 months; DFS not reported separately; only 15 patients in WW group remained disease-free	Group A : 24.2%(15/62) Group B : 25.4%(15/59)	50% in both groups	Median follow-up of 19 months; 2 non-treatment deaths in Group B (traffic accident, cerebral infarction); OS of others not mature	thrombocytopenia : Group A: 24.2% Group B: 33.9% neutropenia: Group A: 11.3% Group B: 5.1%	Sphincter preservation rate: 82.3% (Group A), 86.4% (Group B); Surgical complications: 5% (Group A), 2.9% (Group B)
Li et al. (7)	2025	141/141	pMMR/MSS LARC cT3/4N0 or cTanyN1/2 Tumor ≤12cm from anal verge	Group SCRT-IC: SCRT→ 6 CAPOX + PD-1 inhibitor Group IC-SCRT: 2 CAPOX + PD-1 inhibitor → SCRT → 4 CAPOX + PD-1 inhibitor	Median follow-up of 29 months; DFS not reported separately for both groups	SCRT-IC: 56.9%; IC-SCRT: 53.6%	50% in both groups	Median follow-up of 29 months; OS not reported separately; no treatment-related deaths reported	thrombocytopenia (SCRT-IC: 24.2%; IC-SCRT: 33.9%) neutropenia (SCRT-IC: 11.1%; IC-SCRT: 5.8%)	Sphincter preservation rate: 81.9% (SCRT-IC), 89.9% (IC-SCRT)
Yang et al. (19)	2023	20/18	dMMR/MSI-H LARC cT3-4N0-2M0	PD-1 inhibitor monotherapy → WW/TME	Median follow-up of 25 months; 2-year DFS: 100% in both groups	100% in WW group (7 cases maintained cCR)	84.6% in surgery group (11/13)	Median follow-up of 25 months; 2-year OS: 100% in both groups	irAEs: 35% (7/20); srAEs: 23.1% in surgery group	Tumor regression rate: Median regression 88.89% (WW group), 69.09% (surgery group); No LR or DM
Zhang et al. (17)	2022	33/32	1. dMMR/MSI-H LACRC (cT3-4N0-2M0, AJCC 8th edition); 2. Age 18-75 years; 3. ECOG PS 0-1; 4. No prior radical surgery	PD-1 inhibitor monotherapy (pembrolizumab/sintilimab/tislelizumab), 200mg IV q3w; median 6 cycles (4-10)	Median follow-up of 14 months; No local recurrence/distant metastasis; DFS not reported separately	9.4% (3 LARC cases chose WW)	75.9% in surgery group (22/29)	Median follow-up of 14 months; 100% OS in the entire group	irAEs: nIT phase :37.5% (12/32) , adjIT phase:27.3% (6/22) srAEs: 10.3%	ORR: 100% in surgery group; 2. MPR (≤10% residual tumor): 86.2% (25/29); 3. Median time to response: 3 cycles (for PR)
Chen et al. (18)	2023	17/16	1. dMMR/MSI-H LARC (cT3-4 or N+, AJCC 8th edition); 2. Age 18-75 years; 3. ECOG PS 0-1; 4. No prior antineoplastic treatment	Sintilimab 200mg IV q3w; after initial 4 cycles, assessment: surgery + adjuvant treatment or continued 4 cycles + WW (for patients with cCR)	Median follow-up of 17.2 months; DFS not reported separately; no recurrence	56.25% (9 cases chose WW)	50% in surgery group (3/6)	Median follow-up of 17.2 months; 100% OS in the entire group	Grade ≥3 AEs: 6%	Median time to cCR: 5.2 months; TVRR: 94% of patients had TVRR >20%; R0 resection rate: 100% (6/6)
STELLAR II	2025	218/218	pMMR/MSS LARC; cT3-4/N+; tumor ≤10 cm from anal verge	iTNT group: SCRT → CAPOX/mFOLFOX + Sintilimab ×4; TNT group: SCRT → CAPOX/mFOLFOX ×4; W&W for patients with cCR	Median follow-up 22.1 months; DFS not separately reported; 26 patients in W&W group maintained sustained cCR	iTNT group: 17.3% (19/110); TNT group: 9.3% (10/108)	iTNT group: 43.1% (31/72); TNT group: 20.7% (17/82)	Median follow-up 22.1 months; No treatment-related deaths; OS immature	iTNT group: 34.5% (predominantly thrombocytopenia, diarrhea); TNT group: 19.4%; Grade 3-4 irAEs 5.5%	R0 resection rate: 97.2% (iTNT), 97.6% (TNT); Sphincter preservation rate: ~76% in both groups; iTNT group CR rate 45.5%
TORCH-E	2025	33/33	Early low rectal cancer (T2-3bN0); tumor ≤5 cm from anal verge; pMMR	SCRT → CAPOX + Toripalimab ×4; W&W for patients with cCR, LE for nCR	Median follow-up 24.1 months; DFS not separately reported; 14 patients in W&W group maintained sustained cCR (≥12 months)	48.5% (16/33) (all received W&W)	Overall pCR rate 72.7% (24/33); All 8 patients in LE group achieved ypT0	Median follow-up 24.1 months; Overall OS 100%	33.3% (predominantly thrombocytopenia 27.3%); No treatment-related deaths	Organ preservation rate 60.6%; QoL score in OP group superior to TME group; Local regrowth rate 16.6% (all successfully salvaged by surgery)
UNION	2024	231/231	LARC (cT3-4/N+); ≤10 cm from anal verge; ECOG 0-1	Experimental group: SCRT → Camrelizumab + CAPOX ×2; Control group: LCRT → CAPOX ×2	Follow-up immature; 3-year EFS to be reported	Experimental group: 1.8% (2/113); Control group: 3.4% (4/118)	Experimental group: 39.8% (45/113); Control group: 15.3% (18/118)	Follow-up immature; OS to be reported	Experimental group: 16.8% (predominantly neutropenia 5.3%); Control group: 17.5%; Grade 3-4 irAEs 3.5%	R0 resection rate: 96.2% (experimental), 97.0% (control); Sphincter preservation rate: 94.2% vs 89.9%
SPRING-01	2025	98/98	LARC (cT3-4/N+/EMVI+/MRF+); ≤15 cm from anal verge; ECOG 0-1	Experimental group: SCRT → Sintilimab + CAPOX ×6; Control group: SCRT → CAPOX ×6	Median follow-up 25 months; DFS not separately reported; Progression/metastasis rate: 12% (experimental) vs 25% (control)	Not separately reported; Total CR rate (cCR+pCR): 61.2% (experimental) vs 32.7% (control)	Experimental group: 59.2% (29/49); Control group: 32.7% (16/49)	Median follow-up 25 months; Mortality rate: 6.1% (experimental) vs 8.2% (control)	Experimental group: 33% (predominantly thrombocytopenia 12%); Control group: 35%; Grade 3-4 irAEs 12%	R0 resection rate 100%; Sphincter preservation rate ~72%; No grade ≥3 postoperative complications

Four clinical trials (STELLAR II (27), TORCH-E (28), UNION (29), and SPRING-01 (30)) have further validated the clinical value of neoadjuvant immunotherapy combined with total neoadjuvant therapy (TNT). In terms of efficacy, TNT combined with immunotherapy can significantly improve the pathological complete response (pCR)/clinical complete response (cCR) rate; for instance, the CR rate in the iTNT group of the STELLAR II trial reached 45.5%, which was significantly higher than 25.0% in the TNT group (27). Regarding safety, the incidence of grade 3–4 adverse events ranged from 16.8% to 34.5%, mainly hematological and gastrointestinal reactions, with the incidence of surgical complications comparable to that of traditional regimens, showing good tolerability. Clinically, the 60.6% organ preservation rate in the TORCH-E trial fully highlights the advantages of this strategy in organ protection and long-term disease control (28). These results indicate that immunotherapy combined with neoadjuvant therapy has become one of the core directions of organ preservation strategies for LARC. However, the optimal sequence of different regimens, benefit differences in high-risk populations, and long-term survival data still need to be verified by larger-scale phase III clinical trials.

### 3.3 Impact of interval time on assessment

Different time intervals from the completion of chemotherapy to response assessment are considered potential factors contributing to differences in organ preservation rates among different groups. A standard 6–8 week interval may be insufficient, as some patients develop delayed cCR (13). In the TORCH trial, Group A had a higher cCR rate (43.5% vs. 35.6%), which might be due to the longer interval from the completion of SCRT to re-staging (20). The CAO/ARO/AIO-12 trial also pointed out that the higher pCR rate in Group B might be attributed to the longer median interval between the completion of CRT and surgery compared with Group A (45 days in Group A vs. 90 days in Group B) (24). The CAO/ARO/AIO-16 trial provided more robust evidence, showing that 64% of patients initially assessed as having nCR converted to cCR after 3 months (31). Therefore, appropriately extending the assessment time can identify potential CR patients, improve the organ preservation rate of these patients, and reduce unnecessary surgeries (32).

### 3.4 Considerations in clinical practice and evidence gaps

Although various criteria for defining cCR (e.g., MSKCC, CWWD) exist, there is currently a lack of high-level evidence guiding which standard should be preferentially applied in specific clinical scenarios. The assessment protocols adopted in existing clinical trials are typically tailor-made based on the specific study design, enrolled population, and the available equipment and expertise of the participating centers. For instance, in centers with advanced imaging resources, MRI combined with rectal endoscopy may dominate, whereas in studies emphasizing molecular detection, ctDNA is assigned greater weight. To date, no prospective studies have directly compared the prioritization and predictive efficacy of different assessment criteria within specific clinical subgroups (e.g., low- vs. high-lying tumors, post-immunotherapy vs. post-chemoradiotherapy). Therefore, in clinical practice, the determination of cCR still relies on the comprehensive interpretation of results from multiple available modalities (DRE, endoscopy, MRI, molecular biomarkers) by a multidisciplinary team, taking into account the specific circumstances of the institution. Future research is needed to establish context-based, hierarchical standardized assessment pathways.

## 4 Efficacy assessment methods for the Watch-and- Wait strategy

The accurate assessment of cCR is the cornerstone of the Watch-and-Wait (W&W) strategy. No single modality provides perfect accuracy; therefore, a multimodal approach is essential. The following table summarizes the key techniques, their principles, advantages, and limitations in assessing cCR, setting the stage for the detailed discussion in the subsequent subsections (**Table 3**).

**Table 3:** Multimodal assessment techniques for cCR in LARC: principles, predictive value, and limitations.

Modality	Principle / Key Features	Advantages in Predicting cCR/pCR	Current Limitations	Role in W&W Strategy
**DRE**	- Assesses tumor distance from anal verge, size, texture, mobility, sphincter invasion - Detects blood on glove; confirms low rectal cancer (≤5 cm from anal verge)	- Basic, economical, and non-invasive - Immediate results for initial screening - Complements imaging/endoscopy by evaluating tactile features (e.g., tumor fixation)	- Limited in assessing tumor infiltration depth and lymph node metastasis - Relies on operator experience	**Indispensable Initial Tool** - Core for low rectal cancer assessment - Guides priority of subsequent multimodal evaluations; avoids missed high-risk factors
**MRI**	- Uniform low T2 signal (fibrosis) - No diffusion restriction on DWI - Lymph nodes with short-axis <5 mm	- High spatial resolution - Evaluates rectal wall & mesorectal nodes - mrTRG allows standardized assessment	- Difficulty distinguishing fibrosis from minimal residual tumor - "Pseudoresidue" post-immunotherapy	**Core Tool** Gold standard for morphological re-staging.
**Endoscopy & Biopsy**	- Flat, white scar with telangiectasia - Negative biopsy for tumor cells	- Direct mucosal visualization - Pathological confirmation	- Sampling error - Blind to deep mural residual disease	**Mandatory Verification** Confirms mucosal healing and absence of tumor cells.
**ctDNA**	Detects tumor-derived cell-free DNA in blood.	- High specificity for residual disease - Reflects systemic tumor burden - Allows for dynamic monitoring	- Limited sensitivity (may miss MRD) - Cost and accessibility	**Emerging Game-Changer** Molecular-level "Minimal Residual Disease" monitoring.
**Biopsy Immunoscore** **(IS_b /_ mIS_b_)**	Quantifies density of CD3^+^/CD8^+^ T cells in pre-treatment biopsy.	- Provides insight into tumor immune microenvironment - Independent prognostic value for regrowth risk	- Relies on high-quality biopsy specimen - Not yet standardized - Undergoing validation	**Promising Biomarker** Risk stratification and refinement of patient selection.
**Artificial Intelligence (Radiomics)**	Extracts high-dimensional quantitative features from medical images (e.g., MRI) to build predictive models.	- Objective and quantitative - Potential to identify features beyond human perception	- Requires large datasets for training/validation - Model generalizability and "black box" issue	**Future Direction** Decision support tool to improve diagnostic accuracy.

Bold text in the table highlights key modalities and emphasizes their primary role or significance in the Watch-and-Wait (W&W) strategy.

### 4.1 Digital rectal examination: an indispensable tool

Digital rectal examination (DRE) is the most basic, economical, and indispensable assessment method in clinical practice, especially for low rectal cancer with tumors ≤5 cm from the anal verge. As the primary step of initial evaluation, DRE can intuitively assess the distance from the tumor to the anal verge, size, texture, and sphincter invasion—in the TORCH-E trial, 75.8% of patients with stage T3 disease confirmed tumors ≤5 cm from the anal verge via DRE combined with rigid rectoscopy, providing key evidence for subsequent treatment decision-making (28). During follow-up, the STELLAR II trial performed DRE assessments every 3 months for patients on the watch-and-wait (W&W) strategy, and early local regrowth was detected by DRE in 3 out of 29 patients with clinical complete response (cCR), allowing timely salvage surgery (27). Additionally, the NO-CUT trial incorporated DRE as the initial step of multimodal assessment, combining it with MRI and endoscopy to accurately enroll 26% of cCR patients into the W&W strategy, with a 30-month distant relapse-free survival rate of 95% (33), further confirming the core value of DRE in patient selection.

### 4.2 MRI-based assessment: core tool optimized by multi-modal combination

MRI is regarded as the gold standard for cCR assessment in the W&W strategy. Its high spatial resolution enables clear visualization of rectal wall invasion, mesorectal lymph nodes, and fascial involvement. The use of 3.0T MRI combined with standard sequences is recommended to enhance the visual assessment of treatment response. T2-weighted imaging (T2WI) is the primary method for re-staging rectal cancer, typically performed 6–8 weeks after the completion of nCRT or TNT. The DWI sequence is used to assess residual tumors and tumor regrowth. The addition of MRI tumor regression grading (mrTRG) results in higher specificity and sensitivity in diagnosing pathological complete response (11). Dynamic contrast-enhanced MRI (DCE-MRI) is used to evaluate mucosal blood supply. Miao et al. found that arterial-phase mucosal linear enhancement (MLE) was significantly associated with pCR (34). Most patients with ypT0 showed MLE on arterial-phase T1-weighted imaging (CE T1WI) (38/52, 73%), while MLE was rare in patients with ypT1–4 status (8/187, 4.3%). Additionally, the combination of DWI increased the AUC of pCR prediction to 0.84 (34).

mrTRG is an important tool for efficacy assessment. Based on mrTRG, cCR corresponds to mrTRG 1 (complete fibrosis with no tumor signal), nCR corresponds to mrTRG 2 (predominantly fibrosis with a small amount of intermediate signal), and incomplete response (iCR) corresponds to mrTRG 3-5 (11). A secondary study of the OPRA trial re-stratified 277 participants according to MRI response types and found that patients with MRI-defined cCR had a higher 5-year TME-free survival rate and a lower local regrowth rate compared with patients with nCR (35).

An imaging-histological model based on magnetic resonance imaging exhibits superior classification performance in diagnosing LARC patients who achieve pCR after nCRT compared with experienced radiologists (36). Zhang et al. constructed a radiomics model based on T2WI magnetic resonance imaging (including lymph node boundary contour, signal intensity, size changes before and after treatment, short-axis diameter, and boundary contour) to predict lymph node regression grading in locally advanced rectal cancer after neoadjuvant chemoradiotherapy (37). This model achieved good calibration and discriminative ability in both the training set and validation set (37). It can assist in pre-treatment judgment of whether lymph nodes can achieve complete response, providing important basis for deciding whether to adopt the “W&W” strategy. Yi et al. used T2WI radiomics combined with pre-treatment imaging features and clinicopathological features (cT, cN, CEA) to construct a prediction model. The area under the curve (AUC) values in the training set and test set were 0.91 and 0.87, respectively (38). This model can effectively predict the pathological response of LARC patients to nCRT, particularly whether pCR is achieved. Therefore, this model can serve as a clinical decision-making tool to assist in determining eligibility for the “Watch-and-Wait” strategy.

Beets et al. found that on re-staging MRI after the completion of TNT, patients with lateral pelvic lymph nodes (LLN) ≥ 4 mm had a significantly lower organ preservation rate (P = 0.013) (39), suggesting that LLN ≥ 4 mm after TNT may be a practical threshold for determining eligibility for the W&W strategy. A baseline MRI analysis of the OPRA trial by Williams et al. found that nodal disease, extramural vascular invasion (EMVI), mesorectal fascia (MRF) involvement, and tumor length ≥ 4 cm were independently associated with increased TME requirement (40). In other words, baseline MRI can be used to predict the likelihood of successful W&W. Patients with negative nodal disease, negative EMVI, no MRF involvement, and small tumors may be more suitable candidates for attempting the W&W strategy.

In cases where mucin is present on MRI after neoadjuvant therapy but no tumor residue is detected by endoscopy and physical examination, such patients have traditionally been excluded from the W&W strategy due to concerns about occult residual lesions. Based on this, Judge et al. conducted a study on MRI reports of locally advanced rectal cancer patients who received neoadjuvant therapy at Memorial Sloan Kettering Cancer Center. They found that 20 patients achieved complete response, and among 19 patients who chose the W&W strategy, the local regrowth rate was 21% (4/19), which was comparable to that of the historical control group of patients who underwent surgery (41). Therefore, patients with mucin detected on post-treatment MRI should not be excluded from the W&W strategy if they meet the criteria for cCR.

### 4.3 Endoscopic assessment: morphological verification of clinical response

Endoscopic examination is also an important method for predicting pathological complete response in LARC patients after neoadjuvant chemoradiotherapy. The results of endoscopic examination are correlated with those of digital rectal examination (DRE), magnetic resonance imaging, and CEA detection (< 5 ng/dL). The combined application of these examinations can further improve the accuracy of pathological complete response diagnosis (42, 43). Habr-Gama et al. (44) were the first to define the following endoscopic findings as predictive indicators of treatment response: complete disappearance of the rectal tumor, replaced by a flat, regular white scar with visible telangiectasia on the scar surface. Studies have shown that these findings have high predictive value for pCR (45). Other endoscopic findings, such as ulcers, irregular mucosa, nodules, strictures, or persistent rectal masses, indicate incomplete tumor regression. However, in terms of sensitivity and specificity, these findings cannot be used as reliable indicators for efficacy prediction (46).

### 4.4 Biopsy immunoscore: molecular evaluation based on tumor immune microenvironment

Traditional imaging and endoscopic assessments mainly rely on morphological features and are unable to capture the immune status of the tumor microenvironment. The biopsy-adapted immunoscore (IS_b_) (47) and its modified version (modified IS_b_, mIS_b_) (48) quantify the infiltration characteristics of immune cells in the tumor area, providing molecular-level supplementary evidence for the screening of W&W patients and prognosis prediction. They exhibit unique value particularly in distinguishing cCR from non-cCR and predicting the risk of local regrowth.

It is important to note that both IS_b_ and mIS_b_ are analyzed using formalin-fixed, paraffin-embedded (FFPE) tissue sections obtained from routine diagnostic biopsies, rather than requiring fresh or specially processed biopsy specimens (47, 48). This means that the scoring can be performed on archival pathological material, eliminating the need for additional invasive procedures and enhancing its translational feasibility in clinical practice.

In a 2020 study, El Sissy et al. proposed IS_b_, which uses digital pathology technology to detect the density of total CD3^+^ T cells and CD8^+^ cytotoxic T cells in the tumor area, and confirmed that patients with high IS_b_ (> 70%-100%) had a higher pCR rate after neoadjuvant therapy. IS_b_ was an independent predictor of DFS in surgical patients. In a cohort of 73 W&W patients, patients with high IS_b_ showed no recurrence during follow-up, which was significantly better than that of patients with low IS_b_ (0%-25%). Meanwhile, IS_b_ can optimize cCR assessment. Among patients with ycTNM 0-1, the pCR rate of those with high ISb reached 89%, and the 2-year local regrowth-free rate was 97.3% (47).

In a 2025 study based on the STELLAR trial cohort, Zeng et al. proposed mIS_b_, which modifies the calculation method to the average of the percentile value of CD8^+^cell density and the percentile value of the CD8^+^/CD3^+^ ratio, further improving the efficacy of prognosis assessment. Patients with high mIS_b_ had a 3-year DFS rate of 85.5%, which was significantly higher than that of patients with medium (68.6%) and low (57.2%) grades. The AUC of mIS_b_ for 3-year DFS prediction (0.64) was superior to that of traditional IS_b_ (0.58). In the TNT subgroup, patients with high mIS_b_ had a significantly higher 3-year DFS rate (87.0%) than those with medium and low grades (48).

Currently, IS_b_ and mIS_b_ are mainly used for W&W eligibility screening, risk stratification, and prediction of neoadjuvant therapy resistance. However, both rely on high-quality biopsy specimens, and sampling errors may affect the results. Additionally, the cutoff values need to be validated in larger populations.

### 4.5 Molecular detection: liquid biopsy-driven breakthroughs

ctDNA is released into the peripheral blood from cancer cells due to apoptosis or necrosis. At baseline, ctDNA can be detected in approximately 75% of patients with LARC. After neoadjuvant therapy or surgery, ctDNA can be detected in approximately 15%-20% of patients. The ctDNA clearance rate after neoadjuvant chemoradiotherapy, or the combination of baseline ctDNA with other conventional markers of clinical response, can serve as a promising marker for selecting and monitoring patients undergoing the “Watch-and-Wait” approach (49).

A prospective study by Wang et al. showed that the pCR rate of patients with post-treatment ctDNA clearance was 95.7%, which was significantly higher than that of patients without ctDNA clearance (66.7%) (50). The AUC of the prediction model combining ctDNA and mrTRG reached 0.886, which was significantly higher than that of the mrTRG model alone or ctDNA alone (50). The recurrence risk of patients with positive ctDNA after nCRT was significantly increased, while the 2-year DFS rate of patients with negative ctDNA was 95.5% (50). A Japanese study pointed out that changes in ctDNA (a decrease of ≥ 80%) can be used as a reliable indicator for predicting whether LARC patients achieve pCR after neoadjuvant therapy (51). Additionally, the postoperative ctDNA level is a strong predictor of postoperative recurrence, and the combination with CEA can further improve the prediction accuracy (51). Therefore, ctDNA can serve as an auxiliary decision-making tool for the “Watch-and-Wait” strategy, promoting individualized treatment.

Although the combination of ctDNA and MRI can significantly improve prediction ability, some patients may still be missed by both ctDNA and MRI due to extremely low tumor burden after nCRT (4). However, the 5’-end nucleotide motifs of cell-free DNA (cfDNA) retain strong predictive value. A study by Wang et al. systematically evaluated the value of plasma cell-free DNA (cfDNA) fragmentomic features, particularly the 5’-end motif, in predicting pCR in LARC patients after nCRT (4). Among 103 patients with complete longitudinal samples, the model combining cfDNA 5’-end motif features and mrTRG exhibited excellent predictive efficacy, which was significantly superior to single ctDNA detection or imaging assessment. Notably, even in the population with undetectable ctDNA, this model still maintained good prediction accuracy, suggesting its supplementary value in identifying residual lesions (4).

In addition to ctDNA and cfDNA, gene expression profiles can also predict pCR. Emons et al. developed a 21-transcript classifier that can predict pCR through preoperative biopsy, with a sensitivity of 31%-50%. It has been validated to be robust in 3 independent cohorts and can assist in screening candidates for the W&W strategy (52).

### 4.6 Molecular mechanisms and biomarkers: insights into treatment response and resistance

Radiotherapy and chemotherapy play crucial roles in the neoadjuvant therapy of locally advanced rectal cancer. Radiotherapy can modify the tumor microenvironment by regulating the inflammatory response, thereby enhancing the cytotoxic effects of chemotherapeutic drugs on cancer cells (53). The process of cancer cell killing by chemoradiotherapy involves changes in multiple molecular signals.

Radiotherapy or chemotherapeutic drugs can cause excessive DNA damage, activate the ATM/ATR signaling pathway to trigger cell cycle arrest, and simultaneously interfere with cell division by regulating key factors such as cyclins and cell cycle checkpoint proteins. When the damage reaches a threshold, it induces cancer cell apoptosis (54, 55). Chemotherapeutic drugs can also disrupt the mitotic spindle to prevent cell division, and sustained mitotic arrest can directly lead to cell apoptosis (56). Montori et al. (57) performed NGS analysis on 26 LARC patients (13 with pCR, 13 with pPR) and identified a pathogenic frameshift mutation in the RAD50 gene in one pCR patient. This mutation impairs MRN complex function and enhances radiotherapy sensitivity, making such patients more likely to achieve sustained cCR and favorable candidates for the W&W strategy. Additionally, peripheral blood phosphorylated ATM (p-ATM) levels serve as a functional biomarker: high p-ATM expression post-treatment indicates chemoradiotherapy sensitivity, while low p-ATM expression or ATM gene variants of uncertain significance (VUS, e.g., c.5890A>G) suggests strong DNA repair capacity and potential resistance, requiring further W&W evaluation via combined ctDNA and MRI.

Chemoradiotherapy modulates the Wnt/β-catenin signaling pathway—a key driver in colorectal carcinogenesis and progression. Abnormal activation of this pathway correlates with greater tumor aggressiveness and worse prognosis (56, 58, 59). The inhibition of this pathway by chemoradiotherapy can reduce the migration and invasion abilities of cells, thereby inhibiting cancer cell progression (58). However, caution is needed: the accumulation of nuclear β-catenin may lead to chemoradiotherapy resistance in locally advanced rectal cancer by regulating epithelial-mesenchymal transition (EMT) or cancer stem cell (CSC) properties (60).Miyako et al. (61) studied 60 LARC patients and found that the rate of good histological response to nCRT was significantly lower in the high nuclear β-catenin expression group (score 2+/3+, 37.1%) than in the low expression group (72%, p<0.01). Even with imaging-proven cCR, patients with nuclear β-catenin positivity have an increased risk of local recurrence, making routine W&W strategy not recommended. If adopted, intensified follow-up (once every 2 months for the first 2 years) is required.

The abnormal activation of the PI3K/AKT/mTOR signaling pathway enhances the growth, invasion, and drug resistance abilities of tumor cells (62). DNA damage induced by chemoradiotherapy may activate this pathway, enabling cancer cells to acquire drug resistance by regulating the DNA damage response process (63). Peng et al. (64) studied 97 LARC patients and found that patients with the PTEN:rs12569998 variant had a significantly higher response rate to chemoradiotherapy (adjusted OR = 2.909, P = 0.027), while those with the AKT2:rs8100018 variant had a significantly higher 5-year DFS rate (79.2% vs. 62.3% in wild-type, P = 0.038). Patients carrying PIK3CA or AKT1 mutations have a lower cCR rate after neoadjuvant therapy, requiring priority combination with PI3K inhibitors to optimize treatment regimens before assessing W&W eligibility, avoiding premature non-surgical strategies.

Furthermore, radiotherapy can promote anti-tumor immunity (65). Radiotherapy induces immunogenic cell death (ICD) of tumor cells, releases pro-inflammatory signals such as neoantigens and damage-associated molecular patterns (DAMPs), and promotes the activation of anti-tumor T cells and the accumulation of tumor-infiltrating lymphocytes (TILs) (66). Radiotherapy can induce the upregulation of PD-L1 expression in tumor tissues, increasing sensitivity to immunotherapy (67). The combination of radiotherapy and PD-L1 antibodies can simultaneously modulate the tumor microenvironment, reduce its immunosuppressive effects, and enhance T cell-derived anti-tumor cytokines (68).

The pathological manifestations of the aforementioned molecular mechanisms can serve as supplementary indicators to assist in judging the residual activity of tumors and the risk of drug resistance. For example, even if patients with nuclear β-catenin positivity are assessed as cCR by imaging, they may have an increased risk of local regrowth and require enhanced follow-up surveillance (60).

## 5 Challenges and future directions of the Watch-and- Wait strategy

### 5.1 Current major challenges

#### 5.1.1 Insufficient accuracy of cCR assessment

Existing cCR assessment methods still have limitations, such as false positive or false negative results in imaging examinations. MRI has difficulty distinguishing between fibrosis and minimal residual tumor (with a false negative rate of approximately 20%). Inflammatory reactions after immunotherapy may lead to pseudoprogression and pseudoresidue (11, 19). This may be because it is difficult to distinguish between masses composed of inflammatory cells, necrotic tissue, or fibrous tissue and those composed of tumor cells based on imaging results (19). A study showed that among patients with locally advanced rectal cancer who received neoadjuvant immune checkpoint inhibitor (nICI) therapy, the rates of pseudoprogression and pseudoresidue were 23.1% and 76.9%, respectively (69), and their therapeutic effects were severely underestimated by MRI, endoscopy, and ultrasound.

Therefore, to avoid underestimation of treatment efficacy and unnecessary surgery, a multimodal evaluation strategy is essential for differentiation. First, a longer assessment window (e.g., extended to 6 months) should be permitted to identify delayed clinical complete responses (19). Second, dynamic biomarker monitoring is critical. Clearance or sustained negativity of ctDNA strongly indicates deep tumor regression, while post-treatment ctDNA positivity suggests a high recurrence risk; a continuous decline in serum CEA/CA19–9 can provide corroborative support (69). Third, active acquisition of pathological evidence is key. Repeat biopsies of endoscopically suspicious lesions revealing only inflammatory cells or fibrosis can directly confirm pseudoresidue, while ultrasound-guided biopsy may be considered for deeper lesions (69). Furthermore, cautious interpretation of treatment-related imaging findings is necessary. Newly enlarged lymph nodes may result from immune activation, and the presence of mucin in the tumor bed on post-treatment MRI, when combined with endoscopic and digital rectal examination findings meeting clinical complete response criteria, should not by itself preclude a watch-and-wait strategy (41). Integrating imaging surveillance, dynamic liquid biopsy monitoring, and pathological verification when indicated allows for more accurate discrimination between true and pseudo-residue, thereby optimizing patient selection for organ preservation.

In addition, there are errors in biopsy sampling. Endoscopic biopsy only covers the superficial layer of the intestinal wall, and approximately 15% of deep residual tumors may be missed (3). Furthermore, there are differences in the definition of cCR among different centers, and standardized procedures are lacking, making it difficult to compare data between studies (12).

#### 5.1.2 Lack of long-term follow-up data

The median follow-up time of existing W&W studies is mostly 3–5 years, and long-term data (over 10 years) are scarce. Although short-term data show that the survival rate of the W&W strategy is comparable to that of surgery, the long-term risk of local regrowth and distant metastasis still needs further verification (12). In addition, the impact of the W&W strategy on the long-term quality of life of patients also requires longer-term follow-up assessment.

### 5.2 Future development directions and optimization pathways

Artificial intelligence technology will be deeply integrated into the cCR assessment process. By leveraging radiomics and deep learning algorithms, high-dimensional features can be extracted from multi-modal images to accurately distinguish between post-treatment inflammatory reactions and residual tumors, providing objective and reliable technical support for the initial screening of the W&W strategy (13). PET-MRI combined with targeted probes is expected to improve the detection rate of minimal residual tumors (11), laying a solid foundation for the safe monitoring of W&W patients. The combination of single-cell sequencing and spatial omics can analyze the tumor microenvironment, identify molecular features associated with W&W tolerance, and provide more precise guidance for selecting patients eligible for the W&W strategy (47). By investigating molecular changes during chemoradiotherapy, dynamic gene expression indicators targeting core pathways such as the cell cycle pathway and MAPK signaling pathway can be developed to assist in judging the residual activity of tumors. Additionally, sufficient attention should be paid to the occurrence of pseudoprogression and pseudoresidue during immunotherapy. Studies have shown that most pseudoprogression occurs within the first 3 months of ICI therapy, and a longer observation period is required to evaluate the response to ICI. Therefore, how to develop more assessment methods to reduce the surgical risk of patients without considering the treatment duration, thereby providing patients with more opportunities to preserve organs, is an important direction for future research (20, 69).

In addition to innovations in assessment tools, treatment regimens can be further optimized. As mentioned earlier, immunotherapy is applicable not only to dMMR/MSI-H patients (19) but also to pMMR/MSS patients (20). PD-1 inhibitors combined with TNT can significantly increase the cCR rate. Furthermore, the combination of PD-1 inhibitors with anti-angiogenic agents has also shown favorable therapeutic effects. The NEOCAP study showed that 73% of patients who received combined treatment achieved complete response (68). However, the incidence of adverse events increased significantly, reaching 98% (70). Therefore, future clinical trials are needed to verify its long-term safety.

## 6 Conclusion

As a core non-surgical treatment option for locally advanced rectal cancer, the Watch-and-Wait strategy exhibits tumor control efficacy comparable to that of surgery under the premise of strictly selecting cCR patients, while significantly improving patients’ quality of life. Current evidence indicates that patients who achieve sustained cCR after neoadjuvant therapy and have no high-risk tumor features (EMVI+, MRF+) are ideal candidates for the W&W strategy. Combining MRI with ctDNA detection can significantly improve the accuracy of cCR assessment. Furthermore, intensive follow-up (e.g., every 3–4 months in the first 2 years) coupled with timely salvage surgery can effectively mitigate the risk of local regrowth.

However, the W&W strategy still faces several challenges, including a lack of standardized cCR assessment protocols, the need for validation of predictive biomarkers, and a scarcity of long-term follow-up data. Future efforts should focus on technological innovations, optimized treatment regimens, and high-quality clinical trials to advance the W&W strategy toward precision and standardization, ultimately achieving the goal of patient-centered, individualized care.

## Data Availability

The original contributions presented in the study are included in the article/supplementary material. Further inquiries can be directed to the corresponding authors.

## Glossary

AUC: area under the curve
cCR: clinical complete response
cfDNA: cell-free DNA
CEA: carcinoembryonic antigen
CR: complete response
CRC: colorectal cancer
CRT: chemoradiotherapy
CSC: cancer stem cell
ctDNA: circulating tumor DNA
CWWD: Chinese Watch-and-Wait Database
DAMPs: damage-associated molecular patterns
DFS: disease-free survival
dMMR: deficient mismatch repair
DRE: digital rectal examination
DRFS: distant recurrence-free survival
DWI: diffusion-weighted imaging
EMT: epithelial-mesenchymal transition
EMVI: extramural vascular invasion
ICI: immune checkpoint inhibitor
ICD: immunogenic cell death
INCT-CRT: induction chemotherapy followed by chemoradiotherapy
IS_b_: immunoscore based on biopsy
IWWD: International Watch-and-Wait Database
LARC: locally advanced rectal cancer
LCCRT: long-course chemoradiotherapy
LLN: lateral lymph node
LRFS: local recurrence-free survival
MLE: mucosal linear enhancement
MRI: magnetic resonance imaging
mrTRG: MRI tumor regression grade
MSI-H: microsatellite instability-high
mIS_b_: modified immunoscore based on biopsy
MRF: mesorectal fascia
nCR: near complete response
nCRT: neoadjuvant chemoradiotherapy
nICI: neoadjuvant immune checkpoint inhibitor
OS: overall survival
pCR: pathological complete response
pMMR: proficient mismatch repair
SCRT: short-course radiotherapy
TME: total mesorectal excision
TNT: total neoadjuvant therapy
TIL: tumor-infiltrating lymphocyte
W&W: watch-and-wait
ypTNM: post-neoadjuvant therapy TNM staging.
